# Association of Childhood Exposure to Nitrogen Dioxide and Polygenic Risk Score for Schizophrenia With the Risk of Developing Schizophrenia

**DOI:** 10.1001/jamanetworkopen.2019.14401

**Published:** 2019-11-01

**Authors:** Henriette Thisted Horsdal, Esben Agerbo, John Joseph McGrath, Bjarni Jóhann Vilhjálmsson, Sussie Antonsen, Ane Marie Closter, Allan Timmermann, Jakob Grove, Pearl L. H. Mok, Roger T. Webb, Clive Eric Sabel, Ole Hertel, Torben Sigsgaard, Christian Erikstrup, David Michael Hougaard, Thomas Werge, Merete Nordentoft, Anders Dupont Børglum, Ole Mors, Preben Bo Mortensen, Jørgen Brandt, Camilla Geels, Carsten Bøcker Pedersen

**Affiliations:** 1 Department of Economics and Business Economics, National Centre for Register-Based Research, Aarhus University, Aarhus, Denmark; 2 The Lundbeck Foundation Initiative for Integrative Psychiatric Research, iPSYCH, Aarhus, Denmark; 3 Big Data Centre for Environment and Health (BERTHA), Aarhus University, Roskilde, Denmark; 4 Centre for Integrated Register-based Research, CIRRAU, Aarhus University, Aarhus, Denmark; 5 Queensland Brain Institute, The University of Queensland, St Lucia, Queensland, Australia; 6 Queensland Centre for Mental Health Research, The Park Centre for Mental Health, Wacol, Queensland, Australia; 7 Department of Biomedicine and Center for Integrative Sequencing, iSEQ, Aarhus University, Aarhus, Denmark; 8 Center for Genomics and Personalized Medicine, Aarhus University and Aarhus University Hospital, Aarhus, Denmark; 9 Centre for Mental Health and Safety, Division of Psychology and Mental Health, Manchester Academic Health Sciences Centre, University of Manchester, Manchester, United Kingdom; 10 Department of Environmental Science, Aarhus University, Aarhus, Denmark; 11 Department of Public Health, Aarhus University, Aarhus, Denmark; 12 Department of Clinical Medicine, Aarhus University, Aarhus, Denmark; 13 Department for Congenital Disorders, Statens Serum Institut, Copenhagen, Denmark; 14 Department of Clinical Medicine, University of Copenhagen, Copenhagen, Denmark; 15 Institute of Biological Psychiatry, Copenhagen Mental Health Services, Copenhagen, Denmark; 16 Mental Health Centre Copenhagen, Capital Region of Denmark, Copenhagen University Hospital, Copenhagen, Denmark; 17 Psychosis Research Unit, Aarhus University Hospital Psychiatry, Aarhus, Denmark

## Abstract

**Importance:**

Schizophrenia is a highly heritable psychiatric disorder, and recent studies have suggested that exposure to nitrogen dioxide (NO_2_) during childhood is associated with an elevated risk of subsequently developing schizophrenia. However, it is not known whether the increased risk associated with NO_2_ exposure is owing to a greater genetic liability among those exposed to highest NO_2_ levels.

**Objective:**

To examine the associations between childhood NO_2_ exposure and genetic liability for schizophrenia (as measured by a polygenic risk score), and risk of developing schizophrenia.

**Design, Setting, and Participants:**

Population-based cohort study including individuals with schizophrenia (*International Statistical Classification of Diseases and Related Health Problems, Tenth Revision* code F20) and a randomly selected subcohort. Using national registry data, all individuals born in Denmark between May 1, 1981, and December 31, 2002, were followed up from their 10th birthday until the first occurrence of schizophrenia, emigration, death, or December 31, 2012, whichever came first. Statistical analyses were conducted between October 24, 2018, and June 17, 2019.

**Exposures:**

Individual exposure to NO_2_ during childhood estimated as mean daily exposure to NO_2_ at residential addresses from birth to the 10th birthday. Polygenic risk scores were calculated as the weighted sum of risk alleles at selected single-nucleotide polymorphisms based on genetic material obtained from dried blood spot samples from the Danish Newborn Screening Biobank and on the Psychiatric Genomics Consortium genome-wide association study summary statistics file.

**Main Outcomes and Measures:**

The main outcome was schizophrenia. Weighted Cox proportional hazards regression models were fitted to estimate adjusted hazard ratios (AHRs) for schizophrenia with 95% CIs according to the exposures.

**Results:**

Of a total of 23 355 individuals, 11 976 (51.3%) were male and all had Danish-born parents. During the period of the study, 3531 were diagnosed with schizophrenia. Higher polygenic risk scores were correlated with higher childhood NO_2_ exposure (ρ = 0.0782; 95% CI, 0.065-0.091; *P* < .001). A 10-μg/m^3^ increase in childhood daily NO_2_ exposure (AHR, 1.23; 95% CI, 1.15-1.32) and a 1-SD increase in polygenic risk score (AHR, 1.29; 95% CI, 1.23-1.35) were independently associated with increased schizophrenia risk.

**Conclusions and Relevance:**

These findings suggest that the apparent association between NO_2_ exposure and schizophrenia is only slightly confounded by a higher polygenic risk score for schizophrenia among individuals living in areas with greater NO_2_. The findings demonstrate the utility of including polygenic risk scores in epidemiologic studies.

## Key Points

**Question:**

How is the combination of childhood exposure to nitrogen dioxide and genetic liability for schizophrenia (as measured by a polygenic risk score based on variation in multiple genetic loci and their associated weight for schizophrenia) associated with the risk of developing schizophrenia?

**Findings:**

In this Danish population-based cohort study of 23 355 individuals, increasing polygenic risk score for schizophrenia was correlated with higher childhood nitrogen dioxide exposure. Polygenic risk score for schizophrenia could not fully explain the association between childhood nitrogen dioxide exposure and schizophrenia, and both were independently associated with elevated schizophrenia risk.

**Meaning:**

This study’s findings suggest that schizophrenia risk may be heightened with increasing levels of childhood nitrogen dioxide exposure, but the pathogenic association between these variables remains unclear.

## Teaser

This Danish population-based cohort study uses national registry data to evaluate the association of exposure to nitrogen dioxide in childhood and genetic liability for schizophrenia with the risk of developing schizophrenia.

## Introduction

Schizophrenia is a complex and debilitating neurodevelopmental syndrome with devastating costs to patients, families, and societies. It is a clinically heterogeneous disorder characterized by diverse psychopathologic processes, including cognitive impairment and positive and negative symptoms.^1^ There is significantly increased risk of premature mortality in individuals with schizophrenia, with mean reduction in life expectancy for women of 11.39 (95% CI, 11.06-11.73) years and for men of 13.48 (95% CI, 13.21-13.77) years.^2^ The worldwide prevalence of schizophrenia approaches 1% with an incidence rate of approximately 1.5 per 10 000 individuals annually.^3,4^ Although both genetic and environmental risk factors influence risk of schizophrenia,^5,6,7^ the underlying etiology remains poorly understood.

Air pollution is now the world’s largest single environmental health risk, with 92% of the global population living in places where pollutant levels exceed World Health Organization limits.^8^ Exposure to air pollution is associated with adverse health effects, including elevated mortality risk. Nitrogen dioxide (NO_2_) belongs to a group of gaseous air pollutants produced by road traffic and as a by-product of combustion. Recently, exposure to NO_2_ (as a measure of air pollution) during childhood has been associated with increased risk of psychotic experiences (odds ratio, 1.71; 95% CI, 1.28-2.28)^9^ and schizophrenia (incidence rate ratio [IRR], 1.36; 95% CI, 1.31-1.41).^10^

Schizophrenia is a highly heritable psychiatric disorder with a heritability of 70% to 85% and a concordance rate among monozygotic twins of 40% to 50%.^11,12,13^ Genome-wide association studies (GWASs) indicate that schizophrenia is highly polygenic^12,14^ (ie, multiple genes with additive small effects lead to the disorder). Individually, most of these genes confer a small elevation in risk, but, cumulatively, they have been estimated to explain between one-third and one-half of the genetic variation in liability to schizophrenia.^15,16^ Use of a polygenic score in epidemiologic studies to aggregate these many small effects provides an alternative estimate of the genetic liability to using family history; combined with environmental factors, the polygenic score can help map the schizophrenia risk landscape.^17,18^ Recent Danish studies have shown IRRs of schizophrenia according to a 1-SD increase in polygenic risk score for schizophrenia of 1.46 (95% CI, 1.34-1.60)^19^ and 1.28 (95% CI, 1.19-1.36).^20^

Nitrogen dioxide concentrations vary geographically, with the highest levels in cities, major transport corridors, and coastal land areas near international shipping routes.^21^ Individuals with higher genetic loading for schizophrenia tend to live in more densely urbanized areas,^7,22,23^ possibly owing to intergenerational and intragenerational migration. Although this gene-environment correlation cannot explain the urban residence and later risk of schizophrenia,^23,24^ it highlights the utility of the polygenic risk score in helping to identify novel risk factors. To our knowledge, no studies have previously investigated the association of NO_2_ exposure and genetic liability with the risk of developing schizophrenia.

The Danish longitudinal population-based registers enabled such an approach because they hold information on both residential exposure to NO_2_ and genetic profiles. In a large representative sample of the Danish population, we examined the joint association of childhood NO_2_ exposure and genetic liability for schizophrenia (as measured by a polygenic risk score) with the risk of developing schizophrenia. We investigated whether (1) childhood NO_2_ exposure and polygenic risk score for schizophrenia are correlated; (2) childhood NO_2_ exposure and polygenic risk score for schizophrenia are associated with schizophrenia; (3) a polygenic risk score for schizophrenia changes the association between childhood NO_2_ exposure and schizophrenia; and (4) gene-environmental interaction exists between a polygenic risk score for schizophrenia and childhood NO_2_ exposure.

## Methods

### Data Sources

The study was conducted using data from the Danish Civil Registration System,^25^ which was established in 1968. All individuals living in Denmark are assigned a unique 10-digit civil registration number at birth or immigration date. This number is stored in the registry together with information on sex, date of birth, change of address, date of emigration, vital status, and links to family members. It is used in all Danish registries, allowing for accurate linkage. The Danish Psychiatric Central Research Register^26^ contains information on all discharge diagnoses assigned at psychiatric hospitals in Denmark since 1969 (outpatient contacts registered since 1995). Diagnoses are coded according to the *International Classification of Diseases, Eighth Revision* until the end of 1993, with the *International Statistical Classification of Diseases and Related Health Problems, Tenth Revision* (*ICD-10*) applied thereafter. The integrated Danish air pollution dispersion modeling system, THOR,^27^ has modeled the concentration of air pollution across Denmark since 1979 down to a spatial resolution of 1 km^2^ and an hourly temporal resolution. Since 1981, the Danish Newborn Screening Biobank^28^ has stored residual dried blood spot samples after routine screening in the first days after birth for nearly all infants born in Denmark. The Integrated Database for Longitudinal Labor Market Research and the education registries^29,30^ cover the entire population and contain yearly information from 1980 including income, educational level, and employment status. The Danish Data Protection Agency, the Danish Scientific Ethics Committee, the Danish Neonatal Screening Biobank Steering Committee, and the Danish Health Data Authority approved this study. Studies based exclusively on registry data do not require informed consent from the included individuals according to Danish legislation (Act of Processing Personal Data, Section 10). The Danish Scientific Ethics Committee, in accordance with Danish legislation, has, for this study, waived the need for informed consent in biomedical research based on existing biobanks. This study followed the Strengthening the Reporting of Observational Studies in Epidemiology (STROBE) reporting guideline.

### Study Population

We used data from The Lundbeck Foundation Initiative for Integrative Psychiatric Research (iPSYCH) case-cohort sample, including the precursor studies, to capture all cases of schizophrenia.^31,32^ In brief, based on a study population consisting of all singleton births between May 1, 1981, and December 31, 2005, who were alive and resident in Denmark at their first birthday and had a known mother (N = 1 472 762), a random sample of 30 000 individuals was included. Individuals with a schizophrenia diagnosis as inpatient or outpatient or emergency department contact at a psychiatric hospital were ascertained through the Danish Psychiatric Central Research Register (*ICD-10* code F20). Diagnoses were given from 1994 to 2012 for individuals 10 years and older. Because follow-up commenced at age 10 years, we restricted the data set to individuals born on or before December 31, 2002, and we included only individuals living in Denmark from birth to age 10 years. To ensure having complete information on family history of psychiatric disorder, we retained in the data set only those individuals with a known mother and father and further restricted it to individuals with both parents born in Denmark. The final study population consisted of all individuals meeting the above criteria who had been genotyped. A flowchart describing the inclusion criteria is shown in the eFigure in the Supplement. Statistical analyses were conducted between October 24, 2018, and June 17, 2019.

### Measurement of NO_2_ Exposure During Childhood

Based on the Civil Registration System, we created a complete history of current and former residential addresses including dates of changes in addresses for each individual during the first 10 years of life. These residential addresses were linked with information from the Danish Register on Official Standard Addresses and Coordinates to obtain exact geographical coordinates. The exact locations were aggregated to grid cells of 1 km^2^ before linking with longitudinal information on NO_2_ concentrations. Mean daily NO_2_ concentrations were calculated based on hourly NO_2_ concentrations. We then estimated the mean of all mean daily NO_2_ concentrations between birth and the 10th birthday, accounting for residential changes, and categorized as follows: less than 10, 10 to less than 15, 15 to less than 20, 20 to less than 25, and 25 μg/m^3^ or greater.

### Polygenic Risk Score

DNA obtained from dried blood spot samples from the Danish Newborn Screening Biobank was genotyped using 3 different genotyping chips: Illumina Human 610-Quad BeadChip array, Illumina HumanCoreExome beadchip, or Illumina Infinium PsychArray-24-v.1.1 BeadChip (all, Illumina, Inc). After quality control,^31,33^ the 3 samples comprised 81 561 individuals. For each of the data sets, only the genotyped variants were used for polygenic risk score analysis, and the polygenic risk scores were calculated separately accounting for linkage disequilibrium (LD) using LDpred^34^ for each of them. Summary statistics from GWASs used for the polygenic risk score calculation were obtained from the Psychiatric Genomics Consortium (PGC) discovery sample^12^ comprising 34 600 individuals with schizophrenia and 45 986 control individuals from the PGC GWAS for schizophrenia, excluding the Danish data. For the LD reference, we first randomly sampled 5000 individuals and then excluded individuals who were more than 1 SD from the average 1000 genomes CEU (Utah residents with Northern and Western European ancestry) genotype when projected onto a space spanned by the first 2 principal components in the 1000 genomes (phase 3) and cryptically related individuals (relatedness coefficient [π̂] > 0.05). This subsample of 4456 unrelated individuals of European ancestry broadly reflects the LD pattern of the individuals on which the GWAS summary statistics are based. As parameters for LDpred, we set the LD radius to 100 single-nucleotide polymorphisms (SNPs) for the 2 precursor studies (3570 individuals) and 150 SNPs for the iPSYCH sample (78 003 individuals). The polygenic risk score corresponding to an LDpred *p* parameter (expected fraction of causal variants) of 0.3 was used as the predictive factor for the precursor studies, and LDpred-inf was used for the iPSYCH sample.

The calculated polygenic risk scores were standardized, initially separately for the 3 samples by using the arithmetic mean and SD among controls/subcohort to account for differences in genotyping procedures, and then in the entire sample based on the mean and SD in the subcohort.

### Covariates

We obtained information on age, sex, and parents from the Civil Registration System. Parents were classified hierarchically with a history of schizophrenia spectrum disorder, affective disorder, or any other psychiatric disorder if they had been treated at a psychiatric hospital with these diagnostic categories.^4^ We defined paternal and maternal gross income (in quintiles), their highest educational attainment level (divided into 3 categories: primary school, high school or vocational training, and higher education) and employment status (employed, unemployed, or outside the workforce for other reasons) in the year of cohort members’ 10th birthdays. To prevent confounding due to population stratification (ie, allele frequency differences due to systematic ancestry differences), we conducted principal component analyses using smartPCA of the EIGENSOFT statistical software package, version 6.1.4^35^ and PLINK, version 1.9^36,37^ to generate genomic principal components^38^ based on a relatedness-pruned set of individuals (π̂ ≤0.09) and a subset of SNPs (minor allele frequency >0.001 and pruned for LD [*r*^2^ < 0.05]). All individuals were subsequently projected back onto the principal component space based on their genotypes and SNP loadings.

### Statistical Analysis

Individuals were followed up from their 10th birthday until hospital admission for schizophrenia, emigration, death, or end of follow-up (December 31, 2012), whichever came first. Because cases were oversampled in the case-cohort design, each person’s risk time was weighted according to the case-cohort sampling probability by using inverse probability weighting (Borgan’s estimator II^39^). The weight for cases was set to 1 because all cases were sampled, and the weight for noncases were calculated as the reciprocal of the sampling fraction.

First, we estimated the mean daily childhood NO_2_ exposure and polygenic risk score for schizophrenia cases and noncases after adjustment for age, sex, birth year, parental history of psychiatric disorder, and parental socioeconomic position. The mean polygenic risk score was also adjusted for the first 10 genomic principal components to control for population stratification. The correlation between childhood NO_2_ exposure and polygenic risk score for schizophrenia was estimated using Pearson correlation and linear regression analysis.

We estimated crude and adjusted hazard ratios (AHRs) for schizophrenia with 95% CIs by fitting weighted Cox proportional hazards regression models with robust SEs. The basic model was adjusted for age, sex, and birth year. We then further adjusted for parental history of psychiatric disorder and parental socioeconomic position.

We calculated Nagelkerke *R*^2^ values and transformed them to the liability scale^40^ by including the population prevalence (1%) and the proportion of cases in our sample to thereby quantify the variance explained by NO_2_ exposure, polygenic risk score for schizophrenia, and their interaction. The liability scale has proven better for combining estimates into a comprehensive risk model, particularly for joint models with environmental and genetic risk factors.^41^ We obtained 95% CIs by bootstrapping (n = 10 000).

To improve accuracy of the polygenic risk score for schizophrenia derived in samples with European ancestry, we performed a sensitivity analysis excluding cryptic-related individuals (π̂ >0.2) and ancestral outliers. We selected European ancestry based on an ellipsoid in the space of the first 3 principal components centered and scaled using the mean ± 6 SDs of a subsample with both parents and grandparents born in Denmark. Genomic principal components were then recalculated.

Finally, because the genotyping was done in different batches, we included batches in the model to account for a possible batch effect. Epidemiologic analyses were performed using Stata statistical software, version 15.1 (StataCorp LLC) and SAS statistical software, version 9.4 (SAS Institute Inc). Statistical significance was set at 2-sided *P* < .05. Statistical analyses were conducted between October 24, 2018, and June 17, 2019.

## Results

**Figure 1.**
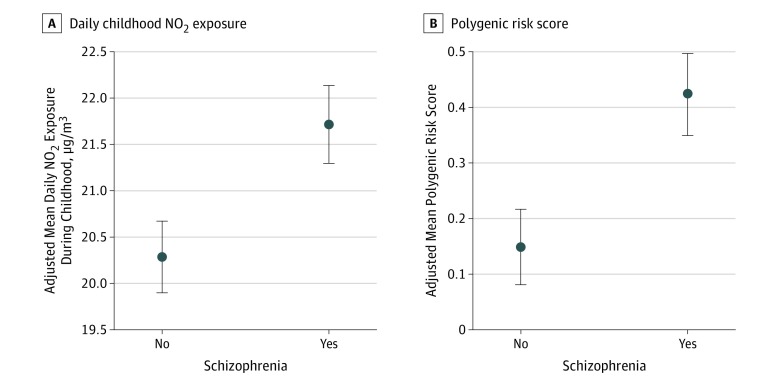
Childhood Mean Daily Nitrogen Dioxide (NO_2_) Exposure and Mean Polygenic Risk Score for Schizophrenia in Participants With and Without Schizophrenia Means and 95% CIs (error bars) were adjusted for age, sex, birth year, parental history of psychiatric disorder, and parental socioeconomic position. Mean polygenic risk score was additionally adjusted for the first 10 genomic principal components.

The case-cohort data set contained 23 355 individuals in total, of whom 11 976 (51.3%) were male. All had Danish-born parents. Description of childhood NO_2_ exposure and polygenic risk score for schizophrenia among 3531 individuals with a schizophrenia diagnosis and 19 907 in the subcohort are given in Table 1. Individuals with schizophrenia had greater childhood NO_2_ exposure (mean [SD], 20.68 [6.22] vs 18.63 [5.92] μg/m^3^/d) and a higher polygenic risk score for schizophrenia (mean [SD], 0.37 [1.04] vs 0.00 [1.00]) (*P* < .001 for both) (Table 1 and Figure 1). Additional characteristics of the population are described in eTable 1 in the Supplement.

**Table 1.** Characteristics of the 23 355 Individuals in the Case-Cohort Study^a^

Characteristics	With Schizophrenia (n = 3531)	Subcohort (n = 19 907)
Childhood exposure to nitrogen dioxide, μg/m^3^/d		
Mean (SD)	20.68 (6.22)	18.63 (5.92)
Category, No. (%)		
<10	86 (2.4)	986 (5.0)
10 to <15	636 (18.0)	5311 (26.7)
15 to <20	1052 (29.8)	5935 (29.8)
20 to <25	738 (20.9)	4304 (21.6)
≥25	1019 (28.9)	3371 (16.9)
Polygenic risk score for schizophrenia		
Mean (SD)	0.37 (1.04)	0.00 (1.00)
Decile, No. (%)		
Lowest decile	202 (5.7)	1990 (10.0)
Second decile	247 (7.0)	1991 (10.0)
Third decile	268 (7.6)	1991 (10.0)
Fourth decile	264 (7.5)	1991 (10.0)
Fifth decile	282 (8.0)	1991 (10.0)
Sixth decile	355 (10.1)	1990 (10.0)
Seventh decile	368 (10.4)	1991 (10.0)
Eighth decile	408 (11.6)	1991 (10.0)
Ninth decile	443 (12.6)	1991 (10.0)
Highest decile	694 (19.7)	1990 (10.0)

^a^ The study base consisted of all individuals born in Denmark from January 1, 1981, to December 31, 2002. Cases were delineated as all individuals who developed schizophrenia from 1994 to 2012. The subcohort members were sampled at random among all individuals in the study base. Therefore, a few individuals belong to both the case group and the subcohort group.

We found a significant correlation between childhood NO_2_ exposure and the polygenic risk score for schizophrenia (ρ = 0.0782; 95% CI, 0.065-0.091; *P* < .001), with the polygenic risk score for schizophrenia explaining 0.61% of the variation in childhood NO_2_ exposure. In an adjusted linear regression analysis, the polygenic risk score was associated with childhood NO_2_ exposure (β = 0.06; 95% CI, −0.01 to 0.13).

We confirmed that both childhood NO_2_ exposure and polygenic risk score were associated with an elevated risk of schizophrenia when included as continuous variables (Table 2). A 10-μg/m^3^ increase in childhood daily NO_2_ exposure was associated with an elevated risk of schizophrenia (AHR, 1.27; 95% CI, 1.19-1.35). A 1-SD increase in polygenic risk score for schizophrenia increased schizophrenia risk (AHR, 1.29; 95% CI, 1.23-1.35). Adjustment for polygenic risk score for schizophrenia slightly attenuated the effect of childhood NO_2_ exposure (AHR, 1.23; 95% CI, 1.15-1.32). This study was designed and conducted from an epidemiologic perspective to follow up a representative sample of native Danish individuals for schizophrenia diagnosis (Table 2). The findings in eTable 2 in the Supplement show that when we further implemented a genetic perspective by excluding cryptically related individuals and ancestral outliers, identical findings were observed (fully adjusted estimate for NO_2_: AHR, 1.25; 95% CI, 1.17-1.34; fully adjusted estimate for polygenic risk score: AHR, 1.29; 95% CI, 1.24-1.35). Also, because batch and birth year are correlated (ρ = –0.85; *P* < .001), adjustment for batches did not change the estimated effect sizes (eTable 3 in the Supplement).

**Table 2.** Risk for Schizophrenia According to Mean Daily NO_2_ Exposure During Childhood and Polygenic Risk Score in a Cohort of 23 355 Individuals

Model	Childhood NO_2_ Exposure, AHR (95% CI)^a^	Polygenic Risk Score, AHR (95% CI)^b^
Model 1^c^	1.26 (1.19-1.34)	1.31 (1.26-1.36)
Model 2^d^	1.27 (1.19-1.35)	1.29 (1.23-1.35)
Model 3^e^	1.23 (1.15-1.32)	1.29 (1.23-1.34)

Abbreviations: AHR, adjusted hazard ratio; NO_2_, nitrogen dioxide.

^a^ The estimate for childhood NO_2_ measures the increased risk of schizophrenia associated with a 10-μg/m^3^ increase in mean daily exposure to NO_2_ during the first 10 years of life.

^b^ The estimate for polygenic risk score measures the increased risk of schizophrenia associated with a 1-SD increase in polygenic risk score. The estimate was also adjusted for the first 10 genomic principal components.

^c^ Adjusted for age, sex, and birth year.

^d^ Adjusted for age, sex, birth year, parental history of psychiatric disorder, and parental socioeconomic position.

^e^ Adjusted for age, sex, birth year, parental history of psychiatric disorder, and parental socioeconomic position. The AHR for childhood daily NO_2_ exposure was also adjusted for the polygenic risk score, and the AHR for polygenic risk score was also adjusted for childhood daily NO_2_ exposure.

**Figure 2.**
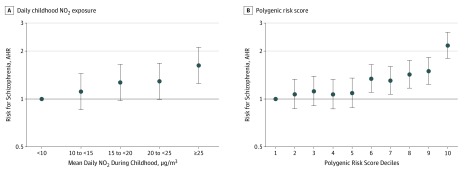
Risk of Schizophrenia by Childhood Mean Daily Nitrogen Dioxide (NO_2_) Exposure and Polygenic Risk Score for Schizophrenia Risk indicates hazard ratio. Hazard ratios are mutually adjusted and further adjusted for age, sex, birth year, the first 10 genomic principal components, parental history of psychiatric disorder, and parental socioeconomic position. Error bars indicate 95% CI.

Categorical analyses confirmed findings of increased risk for schizophrenia with increasing NO_2_ and polygenic risk score as shown in Table 2. There was a dose-response association between schizophrenia risk and increasing levels of both childhood NO_2_ exposure and polygenic risk score (Figure 2). Individuals exposed to daily NO_2_ levels of 25 μg/m^3^ or higher during childhood had a higher risk compared with individuals exposed to daily levels less than 10 μg/m^3^ (AHR, 1.62; 95% CI, 1.25-2.12). Individuals with a polygenic risk score in the highest decile had greater risk compared with those with scores in the lowest decile (HR, 2.17; 95% CI, 1.80-2.62).

Table 3 provides Nagelkerke *R*^2^ and *R*^2^ transformed to the liability scale. Childhood NO_2_ exposure and polygenic risk score (including principal components) explained 0.50% (95% CI, 0.31%-0.75%) of the variance on the liability scale, and polygenic risk score explained 0.82% (95% CI, 0.63%-1.18%) of the variance on the liability scale. Together they explained 1.28% (95% CI, 1.02%-1.72%) in line with additivity. We found no evidence of a significant interaction between childhood NO_2_ exposure and polygenic risk score (estimates for the interaction term: AHR, 1.01; 95% CI, 0.94-1.08; *P* = .82), and including the interaction term in the model did not explain more of the variance.

**Table 3.** Logistic Regression Models Estimating Schizophrenia Risk Associated With NO_2_ Exposure During Childhood and Polygenic Risk Score in a Cohort of 23 355 Individuals^a^

Exposure	Nagelkerke *R***^2^** (95% CI), %	*R*^2^ on Liability Scale (95% CI), %
Childhood mean daily NO_2_ exposure	0.81 (0.50-1.21)	0.50 (0.31-0.75)
Polygenic risk score for schizophrenia	1.32 (1.01-1.90)	0.82 (0.63-1.18)
Childhood mean daily NO_2_ exposure and polygenic risk score for schizophrenia	2.06 (1.65-2.77)	1.28 (1.02-1.72)
Childhood mean daily NO_2_ exposure, polygenic risk score for schizophrenia, and interaction term	2.06 (1.67-2.79)	1.28 (1.03-1.73)

Abbreviation: NO_2_, nitrogen dioxide.

^a^ Schizophrenia risk was estimated singly or jointly with age, sex, birth year, parental history of psychiatric disorder, parental socioeconomic position, and the first 10 genomic principal components.

## Discussion

To our knowledge, this case-cohort study is the first to examine the joint effect of childhood NO_2_ exposure and polygenic risk score for schizophrenia on developing schizophrenia. We found a correlation between childhood NO_2_ exposure and polygenic risk score for schizophrenia, and we confirmed that both were associated with elevated risk of schizophrenia. Also, adjustment for polygenic risk score only slightly attenuated the association of childhood NO_2_ exposure with schizophrenia risk.

The elevated risk for schizophrenia associated with higher NO_2_ levels is consistent with findings of a recent Danish study in which a dose-response association was observed between accumulated exposure to NO_2_ during childhood and subsequent development of schizophrenia (IRR, 1.36; 95% CI, 1.31-1.41 per 10-μg/m^3^ increase in daily NO_2_ exposure).^10^ Our results regarding the polygenic risk score are also consistent with results reported from other Danish studies, in which polygenic risk scores were associated with schizophrenia, for example, Benros et al^19^ (IRR, 1.46; 95% CI, 1.34-1.60) and Sørensen et al^20^ (IRR, 1.28; 95% CI, 1.19-1.36).

Potential biological mechanisms for the association between air pollution and schizophrenia remain uncertain, but air pollutants have been purported to cause inflammation of the tissue of the nervous system, oxidative stress, microglial activation, protein aggregation, subclinical cerebrovascular disease, and disruption of the blood-brain barrier.^42,43^ With the complex clinical features of schizophrenia, it is likely that genetic variation may play a role in determining an individual’s susceptibility to the damaging effects of air pollution.^44^ However, our findings suggest that a polygenic risk score based on common variants related to schizophrenia cannot account for the association between childhood NO_2_ exposure and schizophrenia.

### Strengths and Limitations

A major strength of this study is its use of interlinked data from multiple population-based registers with complete follow-up. The prospectively collected data in these registries minimize the risk of both selection and information bias. Individuals with schizophrenia were ascertained based on contacts with a psychiatric hospital, and schizophrenia diagnoses from the Danish Psychiatric Central Research Register have been shown to be of high validity, with a positive predictive value as high as 97.5%.^45^ In Denmark, treatment is provided through the government health care system free of charge, giving every individual equal health care irrespective of their socioeconomic position. Furthermore, we had complete residential address information for all individuals from birth to age 10 years, which, when linked with daily NO_2_ concentrations in a 1-km^2^ area, yielded an exposure measure for the entire 10-year period. The THOR model is continualy being validated against measurements from Danish monitoring stations and has been shown to reproduce what is measured with high accuracy.^27,46^ Moreover, genotyping information from the large population-based iPSYCH case-cohort sample combined with publicly available GWAS summary statistics for schizophrenia enabled us to calculate a polygenic risk score for schizophrenia.

### Limitations

Our study also has several limitations. By using the schizophrenia diagnoses in the Danish Psychiatric Central Research Register, we did not include individuals diagnosed with schizophrenia who were only treated in primary health care. However, because schizophrenia is a serious psychiatric disorder typically requiring secondary care, this relatively small group is unlikely to significantly change our results. Second, we used residential addresses during childhood to define exposure to NO_2_, but we had no information on day care, school exposures, or travel activities. Third, we used exposure measures from birth to age 10 years; thus, it is possible that other exposure windows may be associated with different findings. Fourth, despite finding a dose-response association between childhood NO_2_ exposure and schizophrenia, it is unknown whether the association can be generalized to settings with lower or higher NO_2_ levels than in Denmark. Fifth, the polygenic risk score aggregates many common risk alleles, but it does not include rare variants and copy number variants,^47^ which may play an important role in the development of schizophrenia.

## Conclusions

This study found that childhood NO_2_ exposure was associated with an elevated risk of schizophrenia that was only slightly explained by a polygenic risk score for schizophrenia. As polygenic scores become more powerful, environmental epidemiology will be able to build more accurate, precise, and comprehensive models of the complex causal pathways between genes, environment, and health outcomes.

## Notes

Supplement. **eFigure.** Flow-chart for the Selection of the Final Study Population **eTable 1.** Baseline Characteristics of the 23,355 Individuals in the Case-Cohort Study **eTable 2.** Risk for Schizophrenia According to Mean Daily NO_2_ Exposure During Childhood and Polygenic Risk Score After Excluding Cryptic-Related Individuals and Ancestral Outliers (N = 22,755) **eTable 3.** Risk for Schizophrenia According to Mean Daily NO_2_ Exposure During Childhood and Polygenic Risk Score Including Batches in the Models (N = 23,355)
