# Supplementary material for: Association of Childhood Exposure to Nitrogen Dioxide and Polygenic Risk Score for Schizophrenia With the Risk of Developing Schizophrenia
Source: JAMA Netw Open. 2019 Nov 1;2(11):e1914401. doi: 10.1001/jamanetworkopen.2019.14401 (PMC6827271; doi:10.1001/jamanetworkopen.2019.14401)
Supplement: Supplement. — eFigure. Flow-chart for the Selection of the Final Study Population eTable 1. Baseline Characteristics of the 23,355 Individuals in the Case-Cohort Study eTable 2. Risk for Schizophrenia According to Mean Daily NO2 Exposure During Childhood and Polygenic Risk Score After Excluding Cryptic-Related Individuals and Ancestral Outliers (N = 22,755) eTable 3. Risk for Schizophrenia According to Mean Daily NO2 Exposure During Childhood and Polygenic Risk Score Including Batches in the Models (N = 23,355) [file jamanetwopen-2-e1914401-s001.pdf]

Supplementary Online Content

Horsdal HT, Agerbo E, McGrath JJ, et al. Association of childhood exposure to nitrogen dioxide and polygenic risk score for schizophrenia with the risk of developing schizophrenia. *JAMA Netw Open*. 2019;2(11):e1914401. doi:10.1001/jamanetworkopen.2019.14401

eFigure. Flow-chart for the Selection of the Final Study Population

eTable 1. Baseline Characteristics of the 23,355 Individuals in the Case-Cohort Study

eTable 2. Risk for Schizophrenia According to Mean Daily NO_2 Exposure During Childhood and Polygenic Risk Score After Excluding Cryptic-Related Individuals and Ancestral Outliers (N=22,755)

eTable 3. Risk for Schizophrenia According to Mean Daily NO_2 Exposure During Childhood and Polygenic Risk Score Including Batches in the Models (N=23,355)

This supplementary material has been provided by the authors to give readers additional information about their work.

eFigure. Flow-chart for the selection of the final study population.

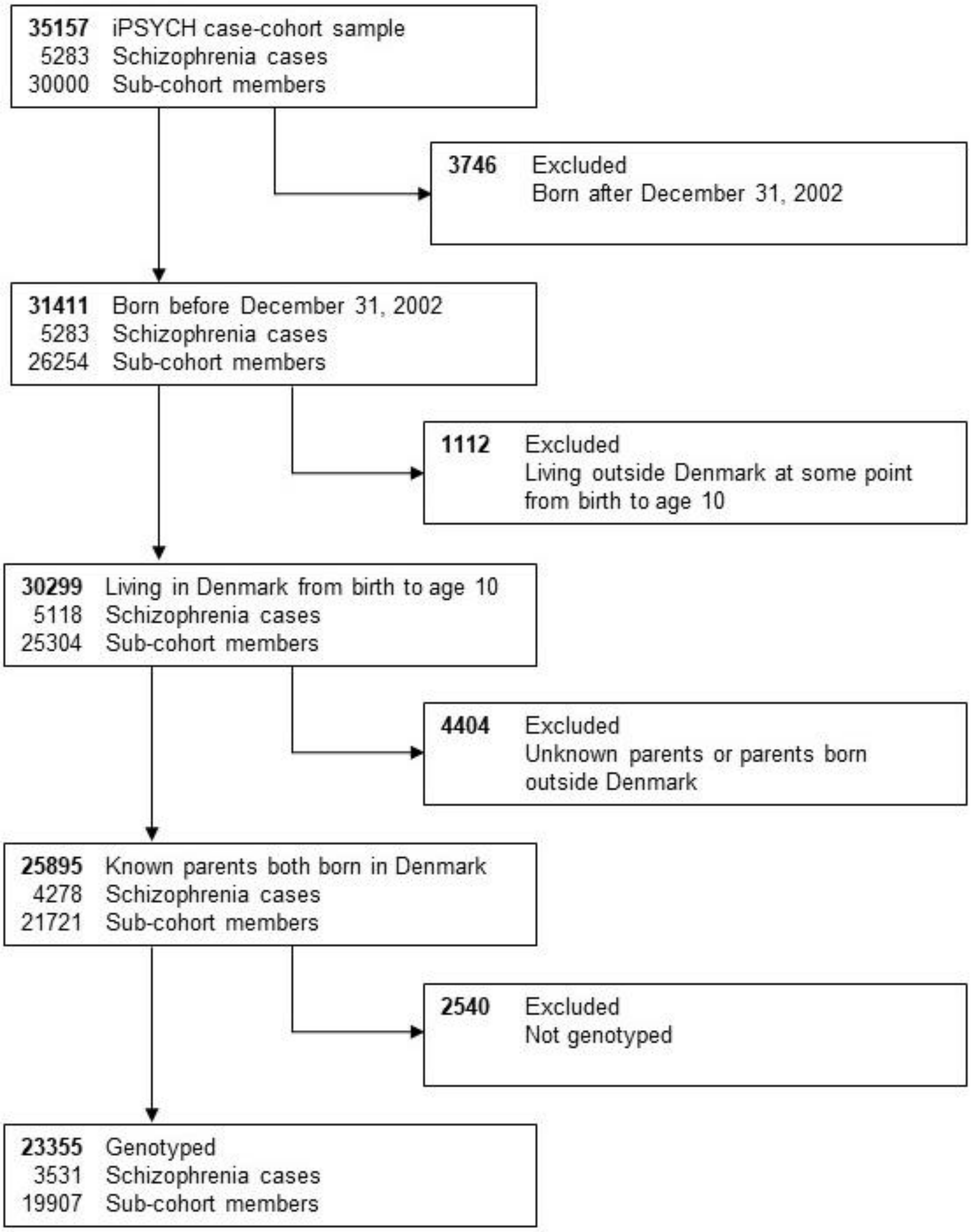

eTable 1. Baseline characteristics of the 23,355 individuals in the case-cohort study.

**Baseline characteristics**	**Schizophrenia (n=3,531)**	**Sub-cohort (n=19,907)**
Gender, No. (%)		
Male	1,926 (54.55)	10,095 (50.71)
Female	1,605 (45.45)	9,812 (49.29)
Parental psychiatric history, No. (%)		
None	2,873 (81.37)	17,939 (90.11)
Any other psychiatric disorder (Other F)	396 (11.21)	1,242 (6.24)
Affective disorders (F30-F39)	97 (2.75)	489 (2.46)
Schizophrenia or related psychosis (F20-F29)	165 (4.67)	237 (1.19)
Father's highest completed educational level, No. (%)		
Primary school	1,298 (36.76)	4,594 (23.08)
High school/vocational training	1,480 (41.91)	9,940 (49.93)
Higher education	667 (18.89)	5,119 (25.71)
Unknown	86 (2.44)	254 (1.28)
Mother's highest completed educational level, No. (%)		
Primary school	1,491 (42.23)	4,995 (25.09)
High school/vocational training	1,209 (34.24)	8,548 (42.94)
Higher education	785 (22.23)	6,257 (31.43)
Unknown	46 (1.30)	107 (0.54)
Father's employment status, No. (%)		
Employed	2,845 (80.57)	18,090 (90.87)
Unemployed	320 (9.06)	867 (4.36)
Outside workforce	366 (10.37)	950 (4.77)
Mother's employment status, No. (%)		
Employed	2,466 (69.84)	16,736 (84.07)
Unemployed	392 (11.10)	1,251 (6.28)
Outside workforce	673 (19.06)	1,920 (9.64)
Father's income, No. (%)		
Lowest quintile	688 (19.48)	2,309 (11.60)
Second quintile	798 (22.60)	3,611 (18.14)
Third quintile	749 (21.21)	4,235 (21.27)
Fourth quintile	687 (19.46)	4,715 (23.69)
Highest quintile	535 (15.15)	4,796 (24.09)
Unknown	74 (2.10)	241 (1.21)
Mother's income, No. (%)		
Lowest quintile	656 (18.58)	2,443 (12.27)
Second quintile	859 (24.33)	4,241 (21.30)
Third quintile	742 (21.01)	4,418 (22.19)
Fourth quintile	684 (19.37)	4,512 (22.67)
Highest quintile	562 (15.92)	4,225 (21.22)
Unknown	28 (0.79)	68 (0.34)

Birth year, No. (%)		
1981-1986	1,470 (41.63)	4,460 (22.40)
1987-1991	1,453 (41.15)	4,710 (23.66)
1992-1996	562 (15.92)	5,075 (25.49)
1997-2002	46 (1.30)	5,662 (28.44)

© 2019 Horsdal HT et al. *JAMA Network Open*.

eTable 2. Risk for Schizophrenia According to Mean Daily NO_2 Exposure During Childhood and Polygenic Risk Score After Excluding Cryptic-Related Individuals and Ancestral Outliers (N=22,755).

**Model**	**Childhood NO_2 exposure, AHR (95% CI)[a]**	**Polygenic risk score, AHR (95% CI)[b]**
Model 1[c]	1.28 (1.20-1.36)	1.32 (1.26-1.37)
Model 2[d]	1.28 (1.20-1.37)	1.29 (1.24-1.35)
Model 3[e]	1.25 (1.17-1.34)	1.29 (1.24-1.35)

Abbreviations: AHR, adjusted hazard ratio; CI, confidence intervals; NO_2, nitrogen dioxide.

[a]The estimate for childhood NO_2 measure the increased risk of schizophrenia associated with a 10 µg/m^3 increase in mean daily exposure to NO_2 during the first 10 years of life.

[b]The estimate for polygenic risk score measure the increased risk of schizophrenia associated with a one standard deviation increase in polygenic risk score. The estimate was additionally adjusted for first 10 genomic principal components.

[c]Adjusted for age, gender, birth year.

[d]Adjusted for age, gender, birth year, parental history of mental disorder, and parental socioeconomic position.

[e]Adjusted for age, gender, birth year, parental history of mental disorder, parental socioeconomic position. Hazard ratio for childhood NO_2 exposure was also adjusted for the polygenic risk score and hazard ratio for polygenic risk score was also adjusted for childhood NO_2 exposure.

Note. This study was designed and conducted from an epidemiological perspective following a representative sample of native Danes (defined as individuals born in Denmark by Danish born parents) for schizophrenia diagnosis (Table 2). The finding in this Table show that when we further implemented a genetic perspective by excluding cryptic-related individuals and ancestral outliers, identical finding were observed.

eTable 3. Risk for Schizophrenia According to Mean Daily NO_2 Exposure During Childhood and Polygenic Risk Score Including Batches in the Models (N=23,355).

**Model**	**Childhood NO_2 exposure, AHR (95% CI)[a]**	**Polygenic risk score, AHR (95% CI)[b]**
Model 1[c]	1.26 (1.19-1.34)	1.31 (1.26-1.36)
Model 2[d]	1.27 (1.19-1.35)	1.31 (1.22-1.41)
Model 3[e]	1.29 (1.14-1.46)	1.30 (1.21-1.39)

Abbreviations: AHR, adjusted hazard ratio; CI, confidence intervals; NO_2, nitrogen dioxide.

[a]The estimate for childhood NO_2 measure the increased risk of schizophrenia associated with a 10 µg/m^3 increase in mean daily exposure to NO_2 during the first 10 years of life.

[b]The estimate for polygenic risk score measure the increased risk of schizophrenia associated with a one standard deviation increase in polygenic risk score. The estimate was additionally adjusted for first 10 genomic principal components and batches.

[c]Adjusted for age, gender, birth year.

[d]Adjusted for age, gender, birth year, parental history of mental disorder, and parental socioeconomic position.

[e]Adjusted for age, gender, birth year, parental history of mental disorder, parental socioeconomic position. Hazard ratio for childhood NO_2 exposure was also adjusted for the polygenic risk sco[illegible]nd hazard ratio for polygenic risk score was also adjusted for childhood NO_2 exposure.

Note. The finding in this Table were expected since batch and birth year are strongly correlated (ρ=-0.85, p<0.001).
